# Association of Smoking with General and Abdominal Obesity: Evidence from a Cohort Study in West of Iran

**Published:** 2017-12-03

**Authors:** Satar Rezaei, Mohammad Hajizadeh, Yahya Pasdar, Mehdi Moradinazar, Behrooz Hamzeh, Farid Najafi

**Affiliations:** ^1^Research Center for Environmental Determinants of Health, Kermanshah University of Medical Sciences, Kermanshah, Iran; ^2^School of Health Administration, Faculty of Health, Dalhousie University, Halifax, Canada; ^3^Department of Nutritional Sciences, School of Public Health, Kermanshah University of Medical Sciences, Kermanshah, Iran; ^4^Department of Public Health, School of Public Health, Kermanshah University of Medical Sciences, Kermanshah, Iran

**Keywords:** Smoking, Obesity, Abdominal obesity, Cross-sectional study, Iran

## Abstract

**Background:** This study aimed to examine the association between smoking and obesity among adults in Kermanshah Province, west of Iran.

**Study design:** A cross-sectional study.

**Methods:** A total of 8822 participants, aged 35-65 yr, form Ravansar Non-communicable Disease (RaNCD) cohort study (2014-2016) were enrolled. Smoking habits were categorized in terms of smoking status (current, former and never smokers) and smoking intensity (light, moderate and heavy). General obesity was defined as body mass index (BMI) ≥30 kg/m2 and abdominal obesity were defined as a waist to hip ratio (WHR) ≥0.9 for men and ≥0.85 for women. Multiple logistic regressions were used to examine the association between general and abdominal obesity with smoking status and smoking intensity while controlling for age, sex, years of education and wealth index.

**Results:** Overall, 12% were current smokers, 8.4% former smokers and 79.6% never smokers. The prevalence of light, moderate and heavy smokers among current smokers was 30.8%, 18%, and 51.2%, respectively. The prevalence of general obesity was 27.6%, while the prevalence of abdominal obesity was 82.3%. The probabilities of general and abdominal obesity for current smokers were lower than never smokers by 34% and 36%, respectively. The probability of abdominal obesity for heavy smokers was 31% lower than light smokers. We did not observe significant associations between smoking intensity and general obesity.

**Conclusions:** Current smokers compared to never smokers were less likely to be obese. The reverse association between smoking and obesity; however, should not be interpreted as a causal relationship.

## Introduction

Smoking and obesity are two leading causes of morbidity and mortality both in developed and developing countries. Several studies have examined the determinants and outcomes of smoking and obesity^1^. It is the fifth leading cause of death worldwide and accounts for 44% and 23% of total cases of diabetes and ischemic heart disease, respectively. Moreover, compared with never smokers, the life expectancy of obese smokers is 13 yr lower^2,3^. Smoking also increases the risk of developing diseases such as cancer, diabetes, and heart disease, which, in turn, leads to a higher level of health expenditure^4-7^.

Although approximately 80% of the current smokers reported intent to quit smoking, only 33% of the smokers attempt to quit smoking. Of smokers who attempted to quit smoking, 75%-80% give up their attempt after 6 months^8-10^. Dependence on nicotine is the most important reason for failing to quit smoking among smokers. Another reason, especially among young women, is related to the concerns about gaining weight upon quitting. There is also a belief, especially among adolescent, that smoking controls body weight and smoking leads to lower body mass index (BMI)^11-13^. Nonetheless, there is no consensus on the association between obesity and smoking and this association is complex and poorly understood. While some studies reported no association between smoking status and BMI^14^, other studies recommended negative association between smoking and BMI^15^.

Few studies examine the association between smoking status and obesity using a large sample of participants in Iran^16^. Thus, the present study aimed to examine the association between smoking (status and intensity) and obesity (general and abdominal) in Kermanshah province, west of Iran using data from the Ravansar cohort study.

## Methods

This study focuses on Ravansar district (a county in Kermanshah Province) located in the western region of Iran close to the Iraqi border. The county’s population is approximately 50000. The population consists mostly Iranian Kurds. The county has 3 urban healthcare centers in the city, and 2 rural healthcare centers and 32 active local healthcare houses in rural areas. We conducted a cross-sectional analysis using baseline data from the Ravansar Non-communicable Disease (RaNCD) cohort study, which is one of 17 centers in the PERSIAN cohort studies (PCS) of Iranian adults that cover a district with both urban and rural areas. All PCSs are prospective epidemiological surveys of the Iranian population established and conducted with the support and coordination of Digestive Disease Research Institute (DDRI) and Ministry of Health and Medical Education (MoHME) to collect valuable information about non-communicable diseases (NCDs) from Iranian adults aged 35-65 yr ^17^.

A total number of 8822 adults (aged 35-65 yr) were surveyed over the period between 2014 and 2016 in the RaNCD cohort study cohort study. The survey collects information on the socioeconomic and demographic characteristics, smoking status and intensity, and anthropometric measures (e.g. general and abdominal obesity) of the participants of the study.

### Smoking behavior

A self-completed questionnaire was used to assess smoking status. Participants were classified based on smoking status as current smokers, former smokers and never smokers. Participants were classified as current smokers if they were smoking at least one cigarette per day and smoked more than 100 cigarettes in their lives. Participants were classified as never smokers if they have never smoked or smoked less than 100 cigarettes in their lifetime. If participants did not smoke regularly or occasionally within the last year and smoke more than 100 cigarettes in their lives, they were classified as former smokers. The current smokers were also categorized based the number of the cigarettes smoked per day (intensity of smoking)^18,19^. The intensity was categorized as light smokers (1–9 cigarettes per day), moderate smokers (10–19 cigarettes per day), and heavy smokers (20 or more cigarettes per day)^18,19^.

### Anthropometric measurements

To measure anthropometric variables of weight, height, BMI, waist circumference (WC), hip circumference (HC) and waist to hip ratio (WHR) participants were asked to remove their shoes, socks, hat, jewelry, accessories (e.g. watch, keys, cell phone), extra layers and heavy clothes. The precision of weight, height, and WC was 0.1 kg, 0.1 cm and 0.1 cm, respectively. WC is measured in light clothing at the narrowest point immediately below the lowest rib and above the iliac crest; HC was measured at the level of the maximum circumference. WHR was calculated as a waist to hip ratio (i.e. WC [cm]/HC [cm]). BMI was computed as follow: weight (kg)/ (height [m] × height [m]) (kg/m^2^). General obesity was defined as BMI≥30. Abdominal obesity was defined as WHR ≥0.9 for men and ≥0.85 for women^20^.

### Statistical analysis

The chi-square test was used to explore the univariate association between smoking status (current, former and never smokers) and smoking intensity (light, moderate and heavy smokers) and the explanatory variables included in the study. Two dependent variables including general obesity and abdominal obesity were included in the study. Multiple logistic regressions were used to examine the association between the general obesity and abdominal (central) obesity with smoking status and smoking intensity while controlling for age, sex, years of education and wealth index. Principle component analysis(PCA) was used to construct household’s wealth index based on their owing assets^21,22^. The wealth score was divided into 5 quintiles as follow: quintile 1= poorest, quintile 2= poor, quintile 3= medium, quintile 4= wealthy and quintile 5= wealthiest. All statistical analysis was performed using Stata statistical software (Version 14.2; Stata Corporation, College Station, TX, USA). *P* -values less than 0.05 was considered to indicate statistical significance.

### Ethical statement

The written informed consent was obtained from each participant after explaining the purpose of the study and was approved by the Ethics Committee of the Deputy of Research at Kermanshah University of Medical Sciences (KUMS.REC.1394.315). Each participant was also informed that s/he had the right to terminate the data collection process at any point. Those who did not provide consent to participate were excluded from the study. Data were collected anonymously and was only used for research.

## Results

Of the total participants included in the study, 52.4% (n=4623) were women. The baseline characteristics of the participants based on smoking status (current, former and never) and smoking intensity (light, moderate and heavy) were reported in Tables 1 and 2. The average age of current smokers, former smokers and never smokers were 47.4 (Standard Deviation [SD]= 7.9), 51.3 (SD=7.9) and 46.6 (SD=8.1), respectively. There was statistical difference between the age groups and smoking status (Chi-square test *P* -value<0.001).

**Table 1:** Characteristics of participants by smoking status in Ravansar cohort, western Iran, 2016

**Variables**	**Current** **smokers(%)** **n=1057(12.0)**	**Former** **smokers(%)** **n=738 (8.4)**	**Never** **S mokers(%)** **n=7027(79.6)**	***P*** **value**
Age groups (yr)				0.001
35-44	457 (43.2)	185 (25)	3608 (51.3)	
45-54	407 (38.5)	282 (38.2)	2135 (30.4)	
55-65	193 (18.3)	271 (36.7)	1284 (18.3)	
Sex				0.001
Male	967 (91.5)	589 (79.8)	2643 (37.6)	
Female	90 (8.5)	149 (20.2)	4384 (62.4)	
Education (yr)				0.001
<3	283 (26.8)	299 (40.5)	2752 (39.2)	
4-7	379 (35.9)	228 (30.9)	2208 (31.4)	
8-11	212 (20.1)	87 (11.8)	820 (11.7)	
>11	183 (17.3)	124 (16.8)	1246 (17.7)	
Body mass index (kg/m^2^)				0.001
<18.5	49 (4.6)	12 (1.6)	90 (1.3)	
18.5-24.9	417 (39.5)	208 (28.2)	1774 (25.2)	
25-29.9	440 (41.6)	344 (46.6)	3053 (43.4)	
>30	151 (14.3)	174 (23.6)	2110 (30.0)	
Abdominal obesity				0.001
Yes	325 (30.8)	131 (17.7)	782 (11.1)	
No	732 (69.2)	607 (82.3)	6245 (88.9)	
Wealth quintile				0.001
1 (Poorest)	1450 (20.6)	130 (17.6)	185 (17.5)	
2	1449 (20.6)	132 (17.9)	183 (17.3)	
3	1373 (19.5)	160 (21.7)	232 (21.9)	
4	1395 (19.8)	155 (21.0)	214 (20.2)	
5 (Wealthiest)	1360 (19.3)	161 (21.8)	243 (23.0)	

**Table 2:** Characteristics of participants according to the smoking intensity in Ravansar cohort, western Iran, 2016

**Explanatory variables**	**Light smokers (%)** **n=326 (30.8)**	**Moderate smokers (%)** **n=190 (17.2)**	**Heavy smokers (%)** **n=541 (51.2)**	***P*** **value**
Age groups (yr)				0.002
35-44	168 (51.5)	86 (45.3)	203 (37.5)	
45-54	106 (32.5)	69 (36.3)	232 (44.9)	
55-65	52 (16.0)	35 (18.4)	106 (19.6)	
Sex				0.001
Male	275 (84.4)	175 (92.1)	517 (95.6)	
Female	51 (15.6)	15 (7.9)	24 (4.4)	
Education (yr)				0.001
<3	80 (24.5)	40 (21.0)	163 (30.1)	
4-7	99 (30.4)	68 (35.8)	212 (39.2)	
8-11	66 (20.2)	46 (24.2)	100 (18.5)	
>11	81 (24.8)	36 (18.9)	66 (12.2)	
Body mass index (kg/m^2^)				0.007
<18.5	9 (2.8)	9 (4.7)	31 (5.7)	
18.5-24.9	106 (32.5)	77 (40.5)	234 (43.3)	
25-29.9	154 (47.2)	75 (39.5)	211 (39.0)	
>30	57 (17.5)	29 (15.3)	65 (12.0)	
Abdominal obesity				0.002
Yes	92 (28.2)	71 (37.4)	216 (39.9)	
No	234 (71.8)	119 (62.6)	325 (60.1)	
Wealth quintile				0.496
1 (Poorest)	53 (16.3)	33 (17.4)	99 (18.3)	
2	62 (19.0)	29 (15.3)	92 (17.0)	
3	68 (20.9)	37 (19.5)	127 (23.5)	
4	59 (18.1)	42 (22.1)	113 (20.9)	
5 (Wealthiest)	84 (25.8)	49 (25.7)	110 (20.3)	

**Figure 1:**
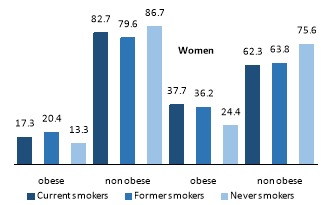
Obesity status by smoking status and sex

Among the participants, 12% (n=1057) were current smokers, 8.4% (n=738) were former smokers and 79.6% (n=7027) were never smokers. The prevalence of light, moderate and heavy smokers in the current smokers was 30.8% (n=326), 18% (n=190) and 51.2% (n=541), respectively. There was also statistically difference between smoking status and sex of participants (Figure 1). Twenty-three percent of the men in the sample (n=967) were current smokers, 14% (n=589) were former smokers and 63% (n=2643) were never smokers. While 2% of women in the sample (n=90) were current smokers, the proportion of former and never smokers in the women sample were 3.2% (n=149) and 94.8% (n=4384), accordingly. The prevalence of general obesity in the overall sample was 27.6%. While the prevalence of general obesity was 16.8% among men; 37.4% of women were obese. The prevalence of obesity was 14.3% among current smokers, 23.6% among former smokers and 30% among never smokers. The overall prevalence of abdominal obesity was 82.3% (94.5% among women and 68.9% among men). Moreover, the prevalence of abdominal obesity was 64.1%, 78% and 85.5% among current smokers, former smokers and never smokers, respectively.

The results of the multiple logistic regressions regarding the effect of smoking status and other explanatory variables included in the study on general and abdominal obesity are presented in Table 3. Current smokers were less likely to be obese than never smokers (odds ratio [OR] for general obesity=0.66; 95% confidence interval [CI], 0.54 to 0.80, and OR for abdominal obesity=0.64; 95 % CI, 0.54-76). The chance of general and abdominal obesity for current smokers was lower than never smokers by 34% and 36%, respectively. Former smokers were more likely to be obese than never smokers (OR for general obesity=1.14; 95% CI, 0.94 to 1.38 and OR for abdominal obesity=1.17; 95% CI, 0.94 to 1.45). These associations were not statistically significant.

**Table 3:** Multiple logistic regression analysis of the association between smoking status and general and abdominal obesity

**Explanatory variables**	**General obesity** **OR (95% CI)**	**Abdominal obesity** **OR (95% CI)**
**Age groups ( yr )**		
35-44	1.00	1.00
45-54	1.07 (0.95, 1.20)	1.05 (0.90, 1.23)
55-65	0.73 (0.63, 0.85)	0.97 (0.80, 1.18)
**Sex**		
Male	1.00	1.00
Female	2.67 (2.37, 3.02)	10.44 (8.64, 12.61)
**Education ( yr )**		
<3	1.00	1.00
4-7	0.99 (0.87, 1.13)	0.98 (0.83, 1.18)
8-11	0.75 (0.63, 0.91)	1.12 (0.89, 1.41)
>11	0.53 (0.43, 0.64)	0.96 (0.76, 1.21)
**Smoking status**		
Never	1.00	1.00
Former	1.14 (0.94, 1.38)	1.17 (0.94, 1.45)
Current	0.66 (0.54, 0.80)	0.64 (0.54, 76)
**Wealth quintile**		
1 (Poorest)	1.00	1.00
2	1.24 (1.06, 1.45)	1.60 (1.30, 1.97)
3	1.34 (1.14, 1.56)	2.13 (1.73, 2.61)
4	1.56 (1.33, 1.83)	2.42 (1.96, 3.00)
5 (Wealthiest)	1.90 (1.59, 2.28)	3.05 (2.43, 3.83)

The effect of smoking intensity and other explanatory variables on general and abdominal obesity are reported in Table 4. There were no significant associations between general obesity with smoking intensity. In addition, heavy smokers were less likely to be abdominal obese than light smokers (adjusted OR 0.69, 95% 0.50-0.97, *P* <0.032).

**Table 4:** Multiple logistic regression analysis of the association between smoking intensity and general and abdominal obesity among current smokers

**Explanatory variables**	**General obesity** **OR (95% CI)**	**Abdominal obesity** **OR (95% CI)**
**Age groups ( yr )**			
35-44	1.00	1.00
45-54	0.56 (0.37, 0.84)	1.10 (0.81, 1.50)
55-65	0.37 (0.19, 0.68)	1.21 (0.79, 1.84)
**Sex**			
Male	1.00	1.00
Female	3.48 (1.78, 6.83)	5.80 (2.65, 12.69)
**Education ( yr )**			
<3	1.00	1.00
4-7	1.15 (0.66, 2.00)	1.13 (0.78, 1.65)
8-11	1.01 (0.53, 1.93)	1.59 (1.00, 2.54)
>11	0.73 (0.36, 1.5)	1.23 (0.74, 2.04)
**Smoking intensity**			
Light	1.00	1.00
Moderate	0.93 (0.56, 1.54)	0.75 (0.49, 1.14)
Heavy	0.78 (0.36, 1.50)	0.69 (0.50, 0.97)
**Wealth quintile**			
1 (Poorest)	1.00	1.00
2	0.73 (0.39, 1.38)	1.47 (0.94, 2.30)
3	1.03 (0.58, 1.85)	1.72 (1.12, 2.63)
4	1.16 (0.65, 2.10)	2.02 (1.29, 3.15)
5 (Wealthiest)	1.53 (0.84, 2.78)	2.62 (1.63, 4.21)

## Discussion

The association between smoking and obesity is poorly documented in Iran^16,23^. In this study, for the first time, we examined the associations between smoking status and intensity with general and abdominal obesity. We found a high prevalence of obesity in the study population; 27.6% (16.8% for men and 37.4% for women) had a BMI≥30 and 82.3% had abdominal obesity (68.9% of men had WHR ≥0.9 and 94.5% women had WHR ≥0.85). Twelve percent of total participants (23% for men and 2% for women) were current smokers, 8.4% (14% for men and 3.2% women) were former smokers and 79.6% (63% men and 94.8% women) were never smokers.

Our results indicated that the general and abdominal obesity for the current smoker were lower than never smokers by 34% and 36%, respectively. Although former smokers were more likely to be obese (both general and abdominal obesity) compared to never smokers, the difference in obesity between these two groups was not statistically significant. These findings were similar to the findings^24^ among Korean adults population. Compared with never smokers, there was no significant association between former smokers and general (adjusted OR = 0.88; 95% CI, 0.70 to 1.10) and abdominal (adjusted OR=0.95; 95% CI, 0.74 to 1.22) obesity. The lower prevalence of obesity for current smokers compared with never smokers that we found was consistent with the findings of several other studies^13,23,25^. For example, the association between sociodemographic and smoking with obesity was investigated among Iranian adult women. There was an inverse association between smoking status and obesity and the current smokers were less likely to be obese than never smokers (adjusted OR = 0.70; 95% CI, 0.50 to 0.99)^23^. “Current smokers were less likely to be obese compared to never smokers (adjusted OR = 0.83; 95% CI, 0.81 to 0.86)^”13^. Smokers' nicotine intake increases the levels of various neurotransmitters and suppresses appetite, and this consequently reduces food intake^26^.

We also assessed the association between smoking intensity with general and abdominal obesity. We found no statistical differences between smoking intensity and general obesity. There was no significant association between general obesity with a number of cigarette smoking per day among Korean adults population^24^. Another study in Indonesia found a nonlinear association between smoking intensity and weight-related outcomes^27^. Our study also indicated that heavy smokers were less likely to be abdominal obese than light smokers. Although we did not observe an association between smoking intensity and general obesity, a study indicated that heavy smoking is associated with higher body weight^28^.

The negative association between smoking and obesity is poorly understood. “Smoking may modify the genetic susceptibility to body fat distribution and overall adiposity” ^29^. Tobacco smoking decreases food intake by reducing appetite and has a negative impact on eating habit of individuals which, in turn, decreases BMI of smokers. Nonetheless, the perceived positive effects of smoking are far less than the benefits obtained from quitting^23^.

This study has several strengths including the large sample size and the use of a well-validated questionnaire for the assessment of smoking status and anthropometrics measurement. Another was the use of anthropometric measurements for weight, height, WC, and HC rather than self-reported data; self-reported data underestimate the BMI of respondents because individuals have a tendency to overestimate their height and underestimate their weight^30^. Our study, however, is subject to some limitations; thus, the findings should be interpreted considering these limitations. First, the present study was a cross-sectional design, which does not allow concluding about the direction of casualty between smoking and obesity. In other words, longitudinal studies are required to establish temporality between explanatory factors and obesity status. Second, although we adjusted for some of the important confounders in our analysis, we did not control for some of the potential confounding factors (e.g., physical activity and energy intake) in our study because this information was not ready for analysis. Third, our study may be subject to the recall bias because the collected data on smoking behavior was self-reported.

## Conclusions

Our study highlighted the association between smoking and obesity. The current smokers were less likely to be obese than their never smoker counterparts. We found statistically significant association between heavy smokers and abdominal obesity. We did not observe any significant associations the number of cigarette smoked per day and general obesity. The reverse association between smoking and obesity, however, should not be interpreted as a causal relationship.

## Acknowledgements

We respect and thank Professor Reza Malekzadeh director of the PERSIAN cohort and Dr Hossein Poustchi executive director of the PERSIAN cohort for helping us to conduct this study in Ravansar.

## Conflict of interest statement

The authors declare that they have no conflict of interest.

## Funding

This work funded by Iran’s Ministry of Health and Medical Education (MoHME) and Kermanshah University of Medical Sciences (KUMS).

## Highlights

- The overall prevalence of current, former and never smokers was 12%, 8.4% and 79.6%, respectively.
- The prevalence of general and abdominal obesity among adults in the west of Iran was 27.6 and 82.3%, respectively.
- The probability of general obesity (BMI ≥30 kg/m2) was 34% lower for current smokers compared to never smokers.
- The probability of abdominal obesity was 36% lower for current smokers compared to never smokers.
- The probability of abdominal obesity was 31% lower for heavy smokers compared to light smokers.
